# German Psychological Literature
*Allgemeine Zeitschrift für Psychiatre und psychisch-gerichtliche Medicin, &c. &c. Unter der Redaction von Damerow Flemming und Roller. Berlin: 1850.


**Published:** 1851-04-01

**Authors:** 


### Art. IV.—GERMAN PSYCHOLOGICAL LITERATURE.*

We are indebted to the accomplished editors of the Berlin "Universal Journal of Psychiatry" for the seventh volume of that ably-conducted periodical. The three numbers or parts which constitute the volume before us fully sustain its high and well-deserved reputation. We proceed to make some extracts from the many very interesting articles which they contain.

The first paper to which we shall refer, is entitled "Pathological Sketches (Darstellungen) characteristic of the various Organs of the Brain and of their Functions; together with an Anatomico-Physiological Introduction." In this ingenious and fanciful article, the author, Dr. G. H. Bergmann, presents us with a selection of detailed and striking cases, from his own long and varied experience, with the two-fold purpose—1st, Of showing how the disorganization or grave injury of the brain affects the various mental operations; and, 2ndly, Of opposing certain views tending in his opinion towards materialism.

Laying it down as an axiom, that a sound basis for physiological and psychological inquiry can be formed only by a persevering study of comparative anatomy, aided by constant investigation into and comparison of the course of each disease, and its peculiar and individual characteristics, with the excellences and deficiencies of the organs of the brain of the individual in whom they are observed, the author proceeds to his first or anatomical section, wherein his inquiries are shown to have been conducted with marvellous minuteness and ability, but in a spirit of speculative assumption which might probably stagger his less metaphysical fellow-labourers on this side the channel.

Dividing the entire hemispheric mass of the brain into what he is pleased to term "*three circles of life*," closely entwined and always acting in unison, and thus "serving as the basis of mental vitality," Dr. Bergmann confidently distributes into these three divisions the various recognised parts of the brain, as well as many hitherto unrecognised, giving to all alike the designation of *organs*, and assigning, in many cases, to each its separate and special function: "and various," says he, "are the abnormities to which these are subject, and various are the mental defects consequent thereupon." To the *cornua* and adjacent parts in the human brain, our author declares that no analogous or corresponding organism has ever been found in the brain of any of the inferior animals, with the exception of a few of the

* Allgemeine Zeitschrift für Psychiatre und psychisch-gerichtliche Medicin, &c. &c. Unter der Redaction von Damerow Flemming und Roller. Berlin: 1850.

monkey tribe. "Here also," he observes, "as in the other parts, the law holds that, in proportion to the number of the fibres, to their development and complete decussation, is also the intelligence, and the ability of motion." Hence it is, he believes, that beasts are deficient in the power of combination, and of moving their limbs artistically. The author points out, as a circumstance worthy of observation, that the power of motion is injuriously affected by a too firm adhesion of the *pia mater*, as well as by its softening or its induration.

To follow Dr. B. through the complicated details of his investigations in cerebral anatomy, would be impracticable in the brief space which can be here afforded. They are interesting from their minuteness, and often amusing from the whimsical neologisms employed in their description. We may mention, however, that he regards the central or middle cavity of the brain as a sort of *camera lucida*, subservient to the *visual* power of the mind. To the reciprocal action of certain parts (organs) of the brain he ascribes that *unity* of visual objects (single vision with two eyes, as Dr. Wells calls it,) which has not hitherto been satisfactorily accounted for: on the individual functions of these organs the author avows that he can only offer surmises, which continued observation alone can verify. The pineal gland, once regarded as the seat of the soul, Dr. Bergmann does not even consider as an organ *per se*, but rather as a mere medium for communicating vitality to the central region of the brain. Lastly, of certain parts, on which the Doctor somewhat gratuitously confers the perception of *musical* harmony, he thus speaks:

"As long," says he, "as *this organ* is still active (though already suffering and defective), and the deranged (irregehender) patient still retains the consciousness of himself and of his torments, he is at issue with life, because life and mind are no longer in unison. He is often seized by a vehement desire, which he can hardly subdue, and which imparts courage even to the pusillanimous and irresolute, to put a stop with his own hand to his psychical anguish and mental discord; but should the organ decay totally, and thus his partial vital activity cease, we then see complete apathy, because the feeling of the disharmony of life exists no longer."

From the manner in which Dr. Bergmann treats his subjects, we are inclined to infer that he is a disciple of Dr. Gall, though the phrenological hypothesis is not directly referred to.

This elaborate and curious article concludes with detailed accounts of *forty* cases of insanity, with the results of their *post-mortem* examinations, which were conducted under the personal direction and observation of this laborious and enthusiastic inquirer. Our warmest thanks are due to Dr. Bergmann for his arduous and persevering researches in the mysterious field of psychical philosophy. His generous exertions cannot be estimated too highly; nor are they less justly appreciated, because we may be sometimes disposed to smile at the fanciful distribution and classification which he has been pleased to adopt, of parts so well known and familiar; nor because we are compelled to (at least) suspend our judgment as to many of the conclusions which he appears to regard as irrefragable.

The same number contains an elaborate article, entitled, "Contributions towards the History of Psychiatry." The writer, Dr. Bird, referring to his preceding contributions, inserted in the fifth and sixth volumes, adds some few other facts, observing, at the same time, that it is a mistaken notion to suppose, with certain French and German psychologists, that mental insanity occurs more frequently among individuals in high life than among persons in humble station. The reverse he affirms to be the case; and that we hear so much about the insanity of persons of high rank, merely because, from their elevated position, their mental state is more frequently observed and recorded, whilst insanity in the lower walks of life escapes notice. In treating of hereditary predisposition to insanity, he points out the royal family of *Hapsburg* as being notoriously subject to this malady, which, it is said, was introduced among them by the marriage of Philip the Fair with Joan, the insane daughter of Ferdinand the catholic and his wife, Isabella of Spain. This dreadful disease continued to run in the blood until the extinction of the family in Spain, in 1700; and, in 1740, (as far as the male line is concerned,) in Austria also. By intermarriage with the Hapsburgers, insanity has been introduced into several other reigning families. A genealogical table of that infected race, presenting a synopsis of considerable interest, is given in the next page.

The following cases of insanity are cited, resulting from despondency or despair:—

Henry IV., emperor of Germany, was excommunicated by Pope Gregory VII. in 1076, whereupon Bishop William, an adherent of the monarch, excommunicated the Pope, at Easter, in the town of Utrecht. Shortly afterwards the bishop was taken ill, repenting his proceedings against the Pope. He fancied he saw spirits of hell by his bedside, eager to snatch his soul. He declared himself lost for ever, and died in confirmed insanity, from despair.

Christian IV., of Denmark.—During the campaign against Tilly, in 1625, this monarch being, at the time, apparently in good health, had the misfortune, on the 20th of July, to fall from his horse into a pit twenty-two feet deep, which was insecurely covered with planks. The horse was killed upon the spot, and the king himself was buried by the

**Ferdinand**, the Catholic king of Aragon, sinks into melancholy, and dies, 1516.

Isabella, queen of Castile, dies 1504.

**Johanna**, heiress of Spain and the Indies, becomes insane in 1555, and dies, aged 76.

Her husband, Philip, duke of Austria (Philip I. of Spain), dies of pulmonitis in 1506.

Charles V., emperor of Germany and king of Spain, is occasionally disturbed in his mind, and dies in 1558.

Philip II., king of Spain, halts between crime and mental derangement. Dies 1598.

Philip III., king of Spain, mind deranged; dies 1621.

Ferdinand I., emperor of Germany, is of sound mind; dies 1564.

Emperor Maximilian II. is sickly, kind, of sound mind. His consort is Maria, daughter of Charles V.

Emperor Rudolph II. dies 1612; descended on both sides from Johanna. Deranged in mind.

Emperor Matthias dies 1619. His sanity doubtful during the latter part of his life.

Leopold I., emperor of Germany. His consort, Margaretha Theresia, was daughter and sister of the two last Spanish Hapsburgers; she died in 1705.

Charles VI., emperor of Germany, died 1740, when the Hapsburg line became extinct. He was of weak intellect, always busy without doing anything. His daughter, Maria Theresa, an intellectual woman, took after her mother, one of the Brunswick family.

Charles, duke of Styria, dies 1590.

Ferdinand II., emperor of Germany, dies 1637.

Ferdinand III., emperor of Germany, dies 1675. His consort, Marianne, was daughter, sister, and aunt of the three last insane Hapsburgers of Spain.

Maria of Austria. She became totally insane, and died in that state in 1581. Her husband was William the Rich. Their son, John William, duke of Cleves, was insane. He died 1609.

---

Anne of Austria, of sound mind; wife of Louis XIII., king of France; dies 1666.

Louis XIV., king of France.

Louis, dauphin of France, dies 1711.

Philip V., king of Spain, the first Bourbon in Spain, becomes insane; dies 1746. The hereditary disposition remains in his family.

Philip IV., king of Spain, was imbecile; died 1616.

Maria Anne of Spain, empress of Ferdinand III., died in 1646.

His queen, Maria Theresa, of sound mind, dies 1683.

Charles II., of Spain, the last descendant of the house of Hapsburg in Spain, was feeble, suffering, and of unsound mind. His mother was daughter of the emperor Ferdinand III.

Margaretha Theresia of Spain, the empress of Leopold I., died in 1673.

mould that had fallen over him, and a considerable time elapsed before he could be extricated. At first he was believed to be dead, and for three days he remained insensible and speechless; however, he was so far recovered by the 7th of August as to resume the command of his army; but, from that time, his mind was no longer sound. At moments when prompt resolution was required, his hesitation was striking. Towards the end of the year the king had a vision, which appeared to strengthen his failing courage. He fancied that he saw Jesus Christ, attired in a purple robe, with a crown of thorns upon his head, and a broken reed in his hand, looking at him with a melancholy countenance, whereupon he regarded himself as the elected champion of the Saviour. However, on the opening of the campaign in 1626, the king's irresolution and mental distraction became still more apparent. He looked pale and emaciated, and became intensely melancholy; he spoke little, and that incoherently. Subsequently, on the restoration of peace, his mind improved somewhat. He died in 1648.

Rudolph II., Emperor of Germany.—This monarch was, both by the paternal and the maternal side, a great grandson of the insane Joan of Spain. Rudolph was fond of Bohemia, and resided at Prague; and his daily-increasing hypochondria rendered any change intolerable. He had a magic mirror, in which he fancied that he saw the most distant objects, and deciphered the most secret thoughts of others, and yet informers and slanderers were his favourite guests in his solitude. He heard mass in a strangely barred oratorium, and would sit for hours in a rigid posture, watching the operations of painters and watchmakers. The easiest means of obtaining access to him was through the protection of a favourite groom, or of some one of his numerous mistresses. His proneness to anger was dreadful. He spent much of his time in his stables, where he was known to have violated women who had come to speak to him on important matters. His bastards inherited much of his wild passions. One of them murdered people in Vienna. Another was himself put to death by the command of his furious father. Rudolph was at last deposed by the imperial princes, who, in 1606, declared him mad. Towards the end of his life, his mental state improved in some degree. He died in 1642.

The Emperor Charles V.—Balzac and other historians speak of the mental disorder into which Charles V. fell *after* his abdication; it is, however, probable that he would never have abdicated had not his mind been already disturbed. According to the memoirs of Du Ribier (vol. ii., p. 747), the reigning Pope, who no doubt was well informed concerning the emperor's state, assured him that Charles had fallen into the malady of his mother Joan. He is known to have assisted at his own mock funeral, and such proceedings certainly bear the stamp of mental aberration. It is said by some that on his death-bed Charles joined the evangelical church. He died September 21st, 1558, aged 57. No historian has taken due notice of this insanity of Charles V., and, consequently, we do not yet possess a good history of that emperor, nor of his age. Every exertion was made to conceal from the world the hereditary insanity of the Hapsburgers, and for this purpose literary Jesuits were employed, and in return the Jesuits were countenanced; thus the mental aberration of the Hapsburgers was the nurse of Jesuitism. Charles had always been of a melancholy temper—he was rarely or never seen to smile, and when later in life this melancholy was deepened by podagra and syphilis, he used to mope for days together, while torrents of tears gave vent to his longing after monastic solitude and death.

Of Philip V. of Spain our author tells us, that after a fit of madness that monarch considered himself as dead; would neither eat nor drink, and even laid down the government. His imbecile son Louis, a youth of 17, dying soon afterwards, the king was again prevailed upon by the most urgent remonstrances of the clergy to resume, or rather, again to lend his name to the government. Philip died in 1746, leaving as his successor his son Ferdinand VI., who terminated his days in a state of insanity.

The principal cause assigned by Dr. Bird why this malady so obstinately clung to the Hapsburgers and the Spanish Bourbons, is the constant intermarriages which prevailed in these families. That several German, French, and Italian princes had married with princesses of these families without introducing hereditary insanity into the blood of their posterity, our author considers as conclusive proof:—First, That the mischief lay in the constant intermarriages of the Hapsburgers among themselves. Secondly, That through marriages with healthy (*i. e.*, sane) families, the predisposition to this dreadful malady is soon removed. This opinion must have been held by the Emperor Charles VI., who refused to ally himself in marriage with a descendant of a Hapsburg princess.

In this number we have also an interesting paper by Dr. L. Spengler on the "Characteristics and Treatment of Demoniacs." It was composed by the Armenian Bishop Œlisæus von Amathunic. This prelate lived in the fifth century, and his views are worthy of notice, as evincing a mind superior to the prejudices of his contemporaries, and far in advance of the age in which he lived. From this curious little work we extract the following passage, remarkable for its quaint mixture of sense and absurdity:—

"Man is liable to pains, having a body subject to all kinds of suffering. Some of these pains can be cured by experienced physicians, others cease of themselves, others again are incurable, causing lasting maladies, and sometimes even death. These evils, however, proceed from without; they originate in the change of the seasons, in excessive heat, violent cold, vitiated air, or damp climate. Others arise from too much eating, too long fasting, and various well-known causes. Some of them resemble demoniacy, especially when the brain has been injured by severe suffering from the stomach; particularly, also, by the increase and decrease of the moon, *which cause an increase and decrease of the brain*, from which divers pains arise. Such people are seen to foam at the mouth, their saliva and mucus are mingled with blood; they have fits, see spectral apparitions, and become insensible. When, however, they are again quieted, their former consciousness returns, and they have no recollection of what happened during their attack. Thus has arisen the general opinion that it is not a disease, but the Devil which tortures them. Some of these people recover their health, but others die of such attacks. In some, pains in the side and back, with various distortions, trembling, shaking of the hands and feet, present a great resemblance to demoniacy. Such fits recur every day at regular hours, sometimes monthly, sometimes only once a year. Hence, the causes of the evil are manifest: many of them are cured by the physicians; but still people call all such sufferers 'demoniacs.' That there are, however, really people who are plagued by impure spirits cannot be denied. Such were especially those that were brought to Christ, many of whom he healed by his mere word, without the application of any physical remedy whatever, using, at the same time, the word by which people are accustomed to call the evil, namely, 'epileptic,' 'lunatic.' Whenever *He* healed epileptic lunatics and demoniacs, he did so as Man and God; for fevers, diseases, epilepsies, and lunacies do not yield to man without medicine, but to God all evils yield and are healed."

In the third number we have an interesting paper by Dr. Willers Lessen, on the "convulsions" of the Jansenists of Paris. These remarkable exhibitions the writer considers as destitute of every trace of supernatural influence, and even of religious enthusiasm; regarding them merely as an expedient adopted by a sinking party, in order to support their faction; similar expedients having been notoriously resorted to by the ascetics, the puritans, and other parties, when placed in a similar position. The opinion pronounced by the sharp-sighted physicians, questioned on the trial of Martha Brossier (in 1599), Dr. Lessen considers as strictly applicable to the hysterical Jansenists, viz., "*multa ficta, pauca à morbo, nihil à spiritu.*"

"After the death of Jansenius, in 1638," says Dr. Lessen, "his adherents entered upon a violent struggle with the jesuits, who were at that time all-powerful at the French court. Among the distinguished men who ranged themselves on the side of the Jansenists were, Arnauld, Pascal, Nicole, &c. They were, moreover, remarkable for their pure morality, but were, for this reason, actively opposed by the Romish clergy, the pope, and the dissolute court. They were persecuted for their opinions, and the pontiff hurled bulls against them, which bulls many of them were compelled to recognise, by Louis XIV., who also, in 1710, destroyed the famous monastery of the Port Royal —the great stronghold of the Jansenists. The struggle became still fiercer in 1713, after the appearance of the bull *unigenitus*, wherein Quesnel's commentaries on the Gospel (a book in great repute with the Jansenists) were condemned. As this work, which was of a purely moral character, contained nothing heretical, all France was now drawn into the controversy; some receiving the papal bull, and thence called 'acceptants;' others rejecting it, and appealing to a future council, and therefore named 'appellants.' In 1730, the parliament of Paris was compelled to recognise the papal mandate. In this extremity, when the Jansenists saw their cause sinking, they had recourse to a series of pretended miracles, which they declared were performed by God himself, in order to bear witness to the justice of their cause. The opportune death of the ascetic François de Paris, in 1727, supplied the 'appellants' with a wonder-working saint, against whose personal morality no objection could be raised. These miracles lasted from 1727 to 1731, without the accompaniment of convulsions, and were discussed in a great many publications of the time. On the 15th of July, 1731, the archbishop of Paris issued his '*Mandement au sujet d'un écrit qui a pour titre* '*Dissertation sur les miracles et en particulier sur ceux qui ont été opérés au tombeau de Mons. Paris, en l'église de St. Médard à Paris, avec la rélation et les preuves de celui qui s'est fait, le* 3 *Novembre*, 1730, *en la personne d'Anne le Franc*, Paris, 1731." In this publication it was proved beyond doubt that, in the case in question, the imputed cure was a fiction, and that the certificates had been procured in a dishonest manner; in consequence of which the worship at the grave was prohibited. This was a sad blow to the 'appellants.' The zealots had now recourse to a new expedient, the management of which was given to the Abbé Bescherant. The violent pains which used to precede the miraculous cures alluded to, served as forerunners to the 'convulsions' which now took their place. The first person by whom they were displayed was Aimée Pivert, who, according to her own account, felt violent pains whilst at the grave, on the 12th of July, 1731. She screamed aloud, her bones crackled, and her whole frame was violently convulsed. These convulsions continued every day, as well in the churchyard as at home, until the period of her perfect recovery, which took place on the 3rd of August following. The next cure attended by convulsions was that exhibited August 2nd, by the wife of Hardouin, a tailor. This woman pretended to have been paralytic and dumb for some eight days before, and was cured on her way home from the tomb. The next cures were, first, that of a deaf and dumb person; then, those of two women; and, at last, the Abbé Bescherant, who had been lame with club-foot from his infancy, fell into convulsions; and, although no cure was visible, yet were the fits declared to be equally miraculous with the cures themselves, and were represented to the public as sanatory efforts of divine origin, tending to restore health; and they were compared with the movements to which a skilful surgeon subjects his patients, in order to restore the use of contracted limbs. At first, the fits came on while the patients were at the grave, but gradually the whole extent of the churchyard, the church itself, and the charnel-houses, became possessed of the healing virtue. For more than six months people crowded to these sights. 'Allons à St. Médard, voyons le miracle que Dieu y fait!' was the watchword. The gate was thrown open before daybreak; portraits, and copies of the life and progress of M. de Paris, were sold there, and the police was employed in keeping order. At last, in January, 1732, an ordonnance of the king appeared, commanding the closing of the little churchyard of St. Médard. This ordonnance, on the attestations of the most eminent physicians and surgeons of Paris, declares the fits in question to be fictitious, and denounces them as calculated to mislead the credulous, to give rise to scandal and riots, and to facilitate theft and licentiousness. The accompanying procés-verbaux were signed by such men as Winslow, La Peyrannie, Le Dran, and Petit. The papers contain the depositions of seven individuals, who, having been examined by a royal commission, declared that their convulsions had been factitious, and several of these witnesses volunteered to imitate them before the commissioners, one of them declaring that he had repaired to the church at the instigation of his confessor. The ordonnance and the report now became the objects of attack by the appellants, the number of the convulsionists increased, each of them acting separately and endeavouring to outdo the others. The churchyard being shut against them, mould from the grave, water from the neighbouring springs, and even the reliquiæ of the sainted Paris, were sufficient to produce the miraculous effects."

The exhibitions now taking place in private houses embraced a wider range, including representations from the history of the sufferings of Christ, others from the life of Paris; prophecies, spiritual discourses, representations of the state of death and of infancy, as well as the grossest indecencies, among which may be classed the so-called "secours," which latter consisted in kind offices which were rendered, generally by *men*, to the female convulsionists, and which, from having originally consisted in merely holding and preventing the pretended invalids from hurting themselves during the fits, had now become atrociously indecent. The term convulsion was now accordingly extended and applied to that state of divine inspiration in which these women pretended to find themselves during the performance of these absurdities. But as many of them were guilty of gross indecencies whilst in convulsions, and many had before been in prison in consequence of immoral conduct, these miracles were suspected even by their adherents. Their own party now split into sections, some considering all these proceedings as gross frauds, others taking opposite views, from which violent controversies ensued. On the 17th February, 1733, another royal ordonnance appeared, prohibiting the convulsionists under penalty of imprisonment, from exhibiting their fits in private houses—forbidding all the king's subjects from attending such exhibitions—and denouncing the fits themselves as the production of disordered imagination and of fraud. But this ordonnance also found opponents, and the pretended sufferers were declared to be orthodox martyrs. The ordonnance was of no avail, and the extravagances increased. At last, in the year 1735, was published by authority the "*Consultation sur les convulsions*," which, on the 7th of January, was signed by some of the most eminent of the appellants. In this publication the convulsions and the "secours" were condemned, and the miracles said to have accompanied them declared to be deceptions and impostures. This consultation created an extraordinary sensation, and called forth a controversy in which the appellants were reproached with inconsistency, inasmuch as they had formerly entertained opinions diametrically opposite on the same subject; whereupon one of them ingeniously declared, "*Quant à ceux qui aussi bien que moi, ont jugé trop favorablement de cette œuvre, nous n'avons pas de peine à avouer que nous nous sommes trompés.*" This was the crisis of the convulsions—the impostors were now frequently unmasked and ridiculed. The miracles thenceforward decreased, and Jansenism itself declined. Meanwhile, discord arose among the convulsionists themselves, and three parties, or factions, became conspicuous. These were the Augustinists, or adherents of Casse, who had assumed the name of Augustin, and pretended to be the forerunner of the prophet Elijah. Secondly. The Vaillantists, or followers of Vaillant, a priest of Tours, who pretended to be the prophet Elijah himself. And thirdly, the "Moderate convulsionists," who condemned the extravagancies of the other two parties. These three factions attacked each other, and thus prepared their mutual ruin: however, the convulsionists lingered on till 1741, or as some authorities say, till the year 1746.

We subjoin a few cases particularizing the symptoms and proceedings of these "malades imaginaires," though few of them indeed deserved so lenient a designation.

The Abbé Bescherant, the originator of these impostures, had a club-foot, which rather disfigured the limb than prevented him from walking. According to his own account, when he saw the miraculous cures, he hesitated whether he should of his own accord present himself to the divine influence or not. At last he determined to wait for a divine direction, first resolving to consider as such, an appeal from Montpellier —accordingly, he soon afterwards received a letter from a cousin of his at Montpellier, wherein it was urged what an effect a cure operated on himself would produce at that town, where he was well known. In consequence of this letter, he laid himself on the grave of Paris on the 25th of August:—subsequently, being ashamed at the great concourse of people who came to witness his exhibition, he wished to withdraw, but did not do so, owing to the convincing arguments of a friend. He went twice a-day to the grave, accompanied by a great number of spectators. Here he was stript of every tightly fitting article of dress, and he lay half naked with his back on the grave, making the sign of the cross, and calling for heavenly assistance. The bystanders at the same time chanted aloud psalms, &c., from large breviaries; upon this he began to shake, to roll his eyes, and make various grimaces; struck the tombstone with his feet, stretched out his arms, jerked out his legs, raised and lowered in quick succession the region of the stomach, and violently shook his head. At the same time his legs trembled, and his stomach expanded. The spectacle lasted each time more than an hour, during which his adherents protected him from all injury: after this his leg was measured to ascertain by how many lines it had become longer; but if all these lines are added together, it will appear that the diseased leg must have exceeded in length the sound one. After each fit he conversed with his friends, and did not appear fatigued, although he felt the want of rest from not going to bed at his usual early hour. After the little churchyard was shut up he continued to visit the church, but had no fits, either there or in the large churchyard, where the police were stationed; but the fits came on in his own residence; at last he was arrested, when the fits ceased altogether, because, as he stated, heaven did not vouchsafe to the gentlemen of St. Lazare to witness such miracles! However, his leg, according to a medical examination and attestation, remained exactly as before, without any improvement.

Another case is that of Folard, who had formerly been an officer and a free-thinker. Every day regularly he had a fit when, in saying vespers, he came to the "Magnificat," when "he falls to the ground, stretches out his arms in the form of a cross, and remains motionless in that posture. Then he sings psalms, and sometimes weeps. Sometimes a loud sound comes out of his ear. Then, again, he suddenly utters single syllables, which some assert to be Sclavonian. Occasionally he suspends himself by the legs from the arm of his chair, and flounders about like a carp. He often claps his hands, and maintains that, when his eyes are open, he is quite in the dark; but that, when he closes them, he is surrounded by brilliant light. He ties a rope round his neck, and then shaking himself, he remains motionless. Towards the end of the fit he sings; and, when all is over, he says, 'I fancy I am singing.'"

Of the meetings of the Convulsionists, a contemporary periodical, the "Journal Historique," an organ of the appellants, gives the following description:

"In their meetings they recite psalms and prayers. When the Convultionist is suddenly seized with a violent fit, she falls on the carpet, rolls about, and becomes convulsed. To relieve her she is beaten, squeezed, hung up, pulled about, and carried up and down by men employed in rendering her these attentions. During these evolutions the girls hold spiritual discourses, sing psalms and hymns, represent the mysteries of Christ, especially his passion, prophecy, and guess secrets. They do not appear fatigued even after having had fits every day for months, or having been beaten with pieces of wood. One girl is said to have swallowed hot coals, another to have devoured bound books; for example, a copy of the Gospels, together with its case!! Another broke stones and marbles with her head!"

The indecencies they were guilty of are thus described: "They assume immodest attitudes; their hair is dishevelled, their feet and legs are bare, the rest of the body carelessly covered; some in the costume of harlequins, or otherwise fantastically dressed, so as to display their limbs to advantage. From their convulsive movements their dress requires to be frequently adjusted, and this often by the hands of a man."

The pretended miracles were, by their adherents, divided into three classes: first, miraculous cures; second, cures attended by miraculous fits; third, fits without cure, which were designated as a divine work. Moreover, the convulsionists possessed the gift of divination. They could read sealed letters; could *smell*, in the streets, the houses in which other convulsionists were residing. They also prophesied, many of them being able to predict the place and hour when others would have fits. One of them could read letters by touching them with his nose, although his eyes were hoodwinked. They also laid claim to the gift of tongues. Some idea of their blasphemies may be formed from the fact, that brother Augustin asserted himself to be sinless; to be the second John the Baptist, the forerunner of Jesus; and, at last, that he was God himself, maintaining that, instead of three, *four* godheads were to be reckoned. In one of his fits he laid himself on the altar, saying, "Let them look at *me;* I am the sacrifice!" Another impostor, brother Hilaire, baptized in fire and blood. Brother Etienne and another, called the Juif Errant, went forth to meet the prophet Elijah, (to whose advent most of their prophecies related,) and became mad on the way. Brother Augustin also went forth to meet a girl of twelve years old, who had prophecied that he would arrive in Paris in the night of November 21st or 22nd, would lodge at the hotel of the Stag, and then repair to the convent at Calvaire. Among their predictions was that of the restoration of the Jews, and of the last judgment, accompanied by eclipses of the sun, and by the appearance of stars, angels, &c.

As a curiosity, we copy a certificate, signed by eleven persons, attesting the following stupendous absurdity:

"Que nous avons vu ce jourd'hui la nommée Marie Sonnet étant en convulsions, la tête sur un tabouret et les pieds sur un autre, les dits tabourets étant entièrement dans les deux côtés d'une grande cheminée et sous le manteau d'icelle, en sorte que son corps était en l'air audessus du feu qui était d'une violence extrême, et qu'elle est restée l'espace de 36 minutes en cette situation en quatre differents reprises, sans que le drap dans laquelle (sic. in orig.) elle étoit enveloppée n'ayant pas d'habits ait brulé, quoique la flamme passât quelquefois audessus."

The blasphemous impudence and indecency of these girls may be inferred from the fact, that one of them having been brought to bed, declared that there was nothing astonishing in the fact that she, a virgin, should, like the mother of God, have been delivered of a child without a father.

We may conclude our extracts from Dr. Lessen's voluminous article, with the case of an amusing impostor who had nearly carried the joke too far. This individual gave out that he intended to crucify himself, being especially moved thereunto by the Spirit. Having attracted great notice by preparations made for months previously, he on the appointed Good Friday saved himself by flight from the grasp of some zealous companions, who, on his refusal to consummate his laudable project, were proceeding to accomplish it for him by a little *douce violence;* and he was afterwards compelled to confess that the whole was a fiction, and had never been suggested by the Spirit.

Among all the German States it is found that Wurtemburg has proportionately the largest number of individuals of imbecile mind. According to a report presented to the government, there were (in 1846) 5000 cretinistic persons, of whom 150 were of the worst description. The neighbouring Grand Duchy of Baden, among a population of 224,300, in 1810 contained but 213, and in 1845, among a population of 1,300,000 souls only 440 cretins. The first German institution for the reception of these sufferers was established in that kingdom in 1835, at Wildberg, in the district of Nagold, and it was supported by voluntary contributions. Since that time, however, several others have been founded, which are partly supported by government, and a great deal has been effected for the education and relief of these unfortunates.

It appears from an article, entitled "Statistics of Mental Derangement and Epilepsy in the Duchy of Anhalt-Gothen, Germany," that the Duchy of Anhalt numbers about 40,000 souls; among these there are—insane, men, 1; women, 10; labouring under fatuity, men, 1; women, 1; imbeciles of various grades, men, 32; women, 27; epileptic, men, 6; women, 2; epilepsy combined with fatuity, men, 1; women, 1. Cured of mental derangement (cure doubtful), men, 3; women, 7; cured of epilepsy, men, 2; women, 1. The total number is 102. Besides these there are 89 persons disturbed in mind, so that out of every 450 inhabitants, *one* is a sufferer from this dreadful scourge.

In the Faroe Islands (Denmark), which contain about 8000 inhabitants, there are seventy insane persons. The large proportion of these unfortunates is generally ascribed to hereditary predisposition to mental derangement, which, as the Germans phrase it, is *at home* in this group of islands.

The commission appointed by the King of Sardinia to inquire into the causes of cretinism, has presented its report, from which it appears that there were malformations in the skulls of all the cretins examined; that their mass of brain is very small, and that there was an entire absence of muscular vigour in all the cases that came under the cognizance of the commissioners; who, moreover, do not consider the *wen* as a necessary concomitant of cretinism. The cretins are, almost exclusively, found in deep and secluded valleys only. Among a population of 3,650,905 souls, in Sardinia, there are 5073 cretins, of whom 2014 have no wen. The assertion of Saussure, that at an elevation of 1000 metres above the level of the sea cretinism ceases, is refuted by the fact, that at the height of 1600 metres above the sea level, the commissioners found 9 per cent. of the population afflicted with goitres and cretinism.

